# Determinants of users’ continuance intention of digital museums: a self-regulation perspective

**DOI:** 10.3389/fpubh.2024.1338387

**Published:** 2024-11-27

**Authors:** Binyuan Zhang, Tingting Jia, Wenhui Zhang

**Affiliations:** ^1^School of Economics and Trade, Henan Polytechnic Institute, Nanyang, China; ^2^School of Digital Media and Art Design, Nanyang Institute of Technology, Nanyang, China; ^3^School of Media, Zhengzhou University of Economics and Business, Zhengzhou, China

**Keywords:** digital museums, self-regulation framework, interaction quality, media richness, information quality, satisfaction, perceived playfulness

## Abstract

Driven by modern technological innovations (virtual reality, augmented reality, mixed reality and interactive 3D, etc.), digital museums open up new modes of user visitation through virtual exhibition halls and interactive technologies, thus bridging the gap between the museums and their users for in-depth communication. This study explores the determinants of users’ continuance intention to use digital museums based on Bagozzi’s self-regulation framework. We found that appraisal factors (interaction quality, media richness, and information quality) were strong predictors of emotional reaction (satisfaction and perceived playfulness). In particular, media richness and information quality had significant effects on both satisfaction and perceived playfulness. However, interaction quality only positively affected satisfaction. Both satisfaction and perceived playfulness positively influence users’ continuance intention to use digital museums. These findings enrich the literature on digital museums, offer new perspectives and supplements to existing research on user behavior in digital museums, thereby assisting developers and operators of digital museums in more effectively designing their digital systems and enhancing user experience.

## 1 Introduction

Prior scholars have conducted investigations that include user behavior characteristics in museums and evaluations of digital technologies, unanimously advocating for a user-oriented approach in digital museums (1–3). Initially, previous studies have evaluated the behavioral attributes of users of digital museums, encompassing aspects like search behaviors (4), visiting motivations (3), their impact on online participation patterns (5), and the emotional and behavioral shifts exhibited throughout user involvement (6). Subsequently, certain scholars have appraised the importance of interactive experiences in digital museums (7, 8). Pei et al. (9) created a comprehensive framework for evaluating user experiences of virtual reality (VR) interfaces in digital museums, and found significant differences existed between the desktop and mobile VR interfaces in user experiences. Lin et al. (10) emphasized the importance of multisensory interactions for enhancing users’ immersive experiences in museums. In research evaluating the connection between novel digital technologies and user behavior, while scholars have explored the effects of augmented reality (AR) and VR devices on user satisfaction (11), behavioral intentions (12), and motivations for recommendations (13) in digital museums, but few scholars have examined the factors affecting users’ continuance intention to use digital museums (14, 15). Considering that the initial adoption of a technology does not always guarantee its commercial success or lasting utilization, user acceptance is merely the first step for an information system, while its ultimate success depends more on continuous usage (16). The value of this discovery has been corroborated by numerous developers within the realms of e-learning systems (17), digital libraries (18), and social recommender systems (19). Therefore, digital museums should understand their users and effectively examine the relationship between technological functions and museum exhibitions, as well as the user experience, to attract more users to continually use their systems (15, 20). As a hot topic in the current academic community, more details are yet to be added on how to enhance users’ continuance intention to use digital museums.

This study addresses the following research question: what and how do determinants collectively affect users’ satisfaction, perceived playfulness, and continuance intention to use digital museums? To respond to this question, the application of Bagozzi’s self-regulation framework is contemplated within this study. Bagozzi’s framework attempts to understand the interrelations between cognitive, affective, and conative variables, presuming that attitudes generate desires, which then lead to an individual’s behavioral intentions, and emphasizing the role of cognitive and emotional self-regulation mechanisms (19). In the research context of digital museums, we revisited the literature pertaining to the factors related to the model. Firstly, in the few studies that assess the continuance intention to use digital museums, Wu et al. (15) found that the persistent intention of individuals to use digital museums is closely related to extrinsic and intrinsic motivational factors. They also discovered that mixed reality (MR) technology can improve the entertainment and usability of digital museums, and this interactive mode is conducive to meeting users’ expectations. Furthermore, a system offering substantial media richness can cater to users’ needs for information access while also enhancing perceived playfulness, thereby becoming a critical determinant of users’ continuous intentions. This perspective has been reiterated in the research conducted by Jiang et al. (11) and Shi et al. (21), where scholars suggest that satisfaction and perceived playfulness are key factors in users’ continuous intention to use digital museum technologies, wherein the pleasure experienced can effectively satisfy hedonic needs, thereby enhancing the continuous intention. Additionally, the high-quality information output has increased the experiential satisfaction of museum visitors, playing an important role in enhancing the perception of playfulness (22). Thus, this study forecasts that the three factors of interaction quality, media richness, and information quality would affect users’ satisfaction and perceived playfulness, thereby affecting their continuous intention to use digital museums.

This research presents several significant scholarly contributions. Firstly, it establishes its theoretical foundation on Bagozzi’s self-regulation framework, using it to examine the appraisal process, emotional reactions, and coping response of users toward digital museums. It specifically investigates how interaction quality, media richness, and information quality impact users’ satisfaction and perceived playfulness, which in turn influence their continuance intention to use digital museums. Its guidance and supporting suggestions provide a new perspective and complement to existing research on user behavior in digital museums. Secondly, the study indicates the critical role of interaction quality, media richness, and information quality as determinants of user satisfaction and perceived playfulness, thereby strengthening the existing literature. Finally, it confirms that both satisfaction and perceived playfulness positively influence users’ continuance intention to use digital museums. These findings are expected to enhance our understanding of user behavior in the context of digital museums, thereby assisting developers and operators of digital museums in more effectively designing their digital systems and enhancing user experience.

## 2 Theoretical background

### 2.1 Digital museums

Generally, digital museums are initiatives that provide users with digital materials and services related to intangible heritage. They use digital tools to transform physical museum collections into data resources, which allows for widespread sharing of valuable information online (23). It is essentially the construction of an information service system around user demands and a straightforward digital exhibition of cultural assets (14).

As informational systems, digital museums foster cultural promotion and knowledge transmission via the internet medium. Previous studies have primarily focused on the characteristics of user behavior in digital museums (2, 9, 15, 21, 24) and the development and evaluation of digital technology (1, 25, 26, 27). Scholars have emphasized that digital museums should adopt a user-oriented approach, exploring how online visitors use museum resources and interact with them (28). Barbieri et al. (1) presented a user-study methodology, for the comparative evaluation of different design alternatives related to the user interaction with virtual museum systems. Venigalla et al. (29) and Khan et al. (30) developed mobile applications associated with the AR Museum to aid diverse museums in enhancing their user experiences. Lypak et al. (25) developed an information system project for a local history museum using augmented reality. While scholars have explored how to design museum interactive devices to improve user experience, it’s important to recognize that user experience depends on more than just the information technology’s functionality. A positive experience not only influences technology adoption but also impacts users’ intention to continue using it, which is crucial for the sustainability of digital museums. Currently, only a few scholars have investigated the factors that affect users’ continuance intention to use digital museums, and more details need to be added (11, 21).

### 2.2 Self-regulation

This study have adopted the self-regulation framework of attitude, intention, and behavior to explore the process of users’ continuance intention to use digital museums. Bagozzi’s framework is significant in that it emphasizes the roles of cognitive and emotional self-regulation mechanisms. It assumes that attitudes generate desires, which then lead to an individual’s behavioral intentions. This process involves three main steps: appraisal, emotional reaction, and coping response (31, 32). Previous studies have confirmed the significant value of the Bagozzi’s framework in consumer behavior research (19, 22). To better apply Bagozzi’s framework in the context of digital museums, we reviewed related information system (IS) literature to identify the factors influencing continued intention in the theoretical model.

Primarily, in user behavior research, information quality is recognized as a crucial element of the appraisal process in Bagozzi’s framework. It was found that incorporating digital technologies such as AR and MR into museums may attract people, boost their attention, and provide users with higher information quality and enjoyable interactive learning experiences (15, 24). In addition, some studies found that a medium that allows sending and receiving rich information in various ways is more likely to be considered valuable and easy to use (14, 21). Digital museums that employ interactive technologies can give visitors a more engaging experience by facilitating their exploration of cultural resources and generating information that can alter the perception of the recipient in a short amount of time. This increases user satisfaction by giving users useful interactive feedback (11). Based on this, we propose interaction quality, media richness, and information quality as appraisal factors.

Furthermore, users will experience different emotional reaction after appraisal. Satisfaction is a key factor in measuring the success and effectiveness of information systems, especially for user-centric digital museums, which can evoke positive emotions in users through high-quality digital interactive experiences (33). According to Moon and Kim (34), perceived playfulness is characterized by three interdependent characteristics, namely enjoyment, curiosity, and focus. This term bears similarities to perceived enjoyment in IS research. As a key factor influencing behavioral intention, scholars have demonstrated in different fields that perceived playfulness has a significant positive effect on users’ continuance intention (15). Therefore, we will introduce perceived playfulness and satisfaction as determinants of emotional reaction, which will in turn affect users’ coping response, such as continuance intention.

**Figure 1:**
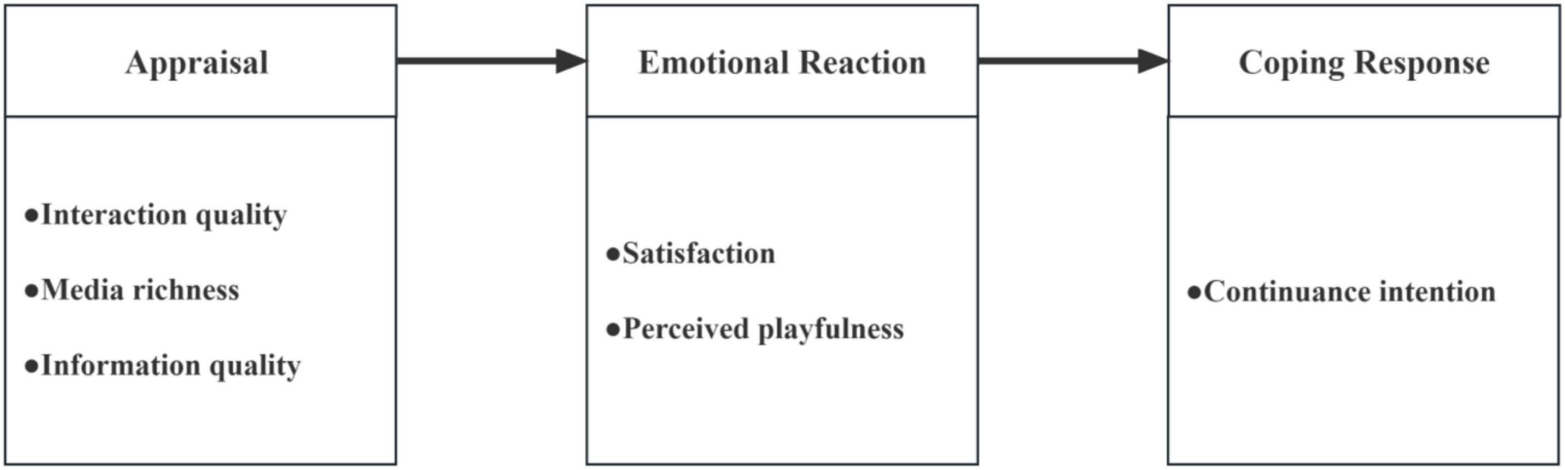
Conceptual construct mappings to Bagozzi’s self-regulation framework.

In summary, this study proposes the research model based on Bagozzi’s self-regulation framework (Figure 1).

## 3 Research model and hypotheses

Figure 1 shows the research model and the hypothesized relationships based on Bagozzi’s Self-Regulation Framework (31). The model takes into account interaction quality, media richness, and information quality as the appraisal factors, users’ satisfaction and perceived playfulness as the emotional reaction factors, and users’ continuance intention as the coping response.

### 3.1 Satisfaction

Satisfaction is conceptualized as a psychological state, emerging from a subjective evaluation based on the discrepancy between actual feelings and expected values (35). In our study, satisfaction refers to the extent to which the user’s experience with digital museums can evoke positive emotions. According to Chiu et al. (36), satisfaction is a common emotional reaction that follows the affective or psychological state of the consumer’s experience. It relates to the consumer’s cognitive evaluation and might have an impact on the consumer’s coping response.

The significance of the correlation between satisfaction and continuance intention has been demonstrated in multiple IS studies (37, 38). For instance, Wu et al. (15) discovered a positive correlation between user satisfaction and the intention for ongoing use in their research on digital museum user acceptance. So, the success of digital museums depends on user requirements, with user satisfaction being a critical part. If users are not happy with the process of acquiring information through digital museums, they may interrupt or transfer to other ways. In line with Bagozzi’s framework, satisfaction, as a significant factor of emotional reaction, affects users’ coping response. Accordingly, we hypothesized:

H1: Satisfaction has positive effects on continuance intention.

### 3.2 Perceived playfulness

In addition to user satisfaction, perceived playfulness is an emotional reaction element in our study model that influences users’ continuance intentions. Perceived playfulness encompasses the inherent beliefs or motivations that emerge from an individual’s interaction with the environment, coupled with the degree of pleasure experienced during the use of a product or service (39). In this study, perceived playfulness is defined in three dimensions, comparable to the idea in information system studies: curiosity, attention, and enjoyment or fun (34).

Preliminary research suggests that perceived playfulness is vital in elucidating user adoption of novel technologies (40). For example, studies in virtual learning (41) and AR digital museums (14) have confirmed that a higher level of perceived playfulness is associated with a strong intention to continue use. Additionally, Wojciechowski and Cellary (42) observed that the software interface design of virtual reality systems has a positive impact on arousing learners’ interest, with studies affirming a positive link between perceived playfulness and the intention of use. Digital museums possess traits such as immersion, interactivity, and intuitiveness, which heighten user interest. By diverging from conventional museum tours, users will generate more positive emotional reaction (like pleasure and curiosity), which in turn inspire behavioral intentions. Hence, we hypothesized:

H2: Perceived playfulness has positive effects on continuance intention.

### 3.3 Interaction quality

While the precise definition of interactivity remains elusive in academic discourse, its role as a key metric in evaluating the success of online information systems is widely acknowledged (43). Interactivity is a multifaceted concept, which can be broadly classified into three categories: user-to-user, user-to-content, and user-to-system interactions (44). Zhao and Lu (45) indicate that interaction quality focuses on measuring the quality provided by service providers. Given that this research scrutinizes the interaction between users and digital museum information systems, interaction quality is delineated as the user’s measurement of the interactive quality provided by digital museum service providers.

Past research on IS suggests that interaction quality may lead to the formation of psychological and behavioral intentions. Beuckels and Hudders (46) found that the interaction quality of a website makes consumers pay positive attention and endeavor to process information, ultimately having a positive effect on attitudes and behavioral intentions. Rodríguez and Meseguer (47) emphasized the importance of the interactivity of digital technology for the formation of students’ psychological images in their research on e-learning. Park and Yoo (48) found that when consumers use shopping websites with AR features, perceived interactivity influences psychological imagery, which in turn affects consumer attitudes and behavioral intentions. Based on this, we reasonably believe that commendable interaction quality may help users form a positive emotional reaction to digital museums by increasing their satisfaction and perceived playfulness. Therefore, we hypothesize that in our model, there is the following relationship between interaction quality and emotional reaction factors:

H3-1: Interaction quality has positive effects on satisfaction.

H3-2: Interaction quality has positive effects on perceived playfulness.

### 3.4 Media richness

In the contemporary world, information of knowledge have become increasingly important, with major service organizations using technological means such as websites to provide users with rich information (49), museums included. Media richness, also termed information richness, denotes the capacity of a medium to generate information that can modify the receiver’s comprehension within a given time frame (50). Feedback capability, multiple cues, channels used, personalization and language variety are among its evaluation criteria (51).

Recently, a growing number of studies have used these four qualities to analyze users’ perceptions of the information-carrying capacity of services or systems, with the goal of studying the link between media richness and other notions. For example, studies have shown that media richness influences user behavior and decision-making in virtual store environments (52), the choice of communication media (53), and the effectiveness of digital marketing channels (54). Wu et al. (15) identified media richness as a critical factor in determining confirmation, perceived ease of use, and perceived usefulness.

In the context of digital museums, which offer users a plethora of information through images, texts, audio, videos, and interactive virtual experiences, the accessibility and engagement surpass that of traditional museums. Based on this, it can be reasonably hypothesized that the employment of rich media forms in museums could potentially enhance user satisfaction and perceived playfulness. Thus, this study proposes the following hypothesis:

H4-1: Media richness has positive effects on satisfaction.

H4-2: Media richness has positive effects on perceived playfulness.

### 3.5 Information quality

An important factor in determining information success is information quality, according to DeLone and McLean’s revision of their D&M IS success model (55). The accurate, reliable, and appropriate information that an information system provides to its users is referred to as information quality. Additionally, it encompasses characteristics of information like its utility, promptness, and the comprehensiveness of its sources (56). The importance of information quality in digital museums is particularly emphasized, as digital museums primarily function to facilitate the public’s convenient access to extensive information on renowned collections in an enjoyable and relaxed way, aiming to enhance appreciation and learning ability.

Prior studies have increasingly acknowledged the critical role of information quality in the efficacy of IS (57). For instance, tom Dieck et al. (58) argue that AR applications in art museums can provide more detailed information about paintings, allowing visitors to gain a deeper understanding of the artwork, thereby fostering a more positive attitude. This indicates that if digital museums ensure they provide high-quality information, users will perceive the museum as capable of fulfilling their needs and are more likely to feel content with it. Furthermore, research indicates that visitors using AR guides are more attentive and involved with the artworks in the museum than those using audio guides or none at all and, their perceived enjoyment and learning efficacy are also improved (59). This means that providing users with accurate, complete, and diverse information can create a more focused and enjoyable touring experience, which is likely to affect the continuance intention of users. Therefore, we hypothesized:

H5-1: Information quality has positive effects on satisfaction.

H5-2: Information quality has positive effects on perceived playfulness.

**Figure 2:**
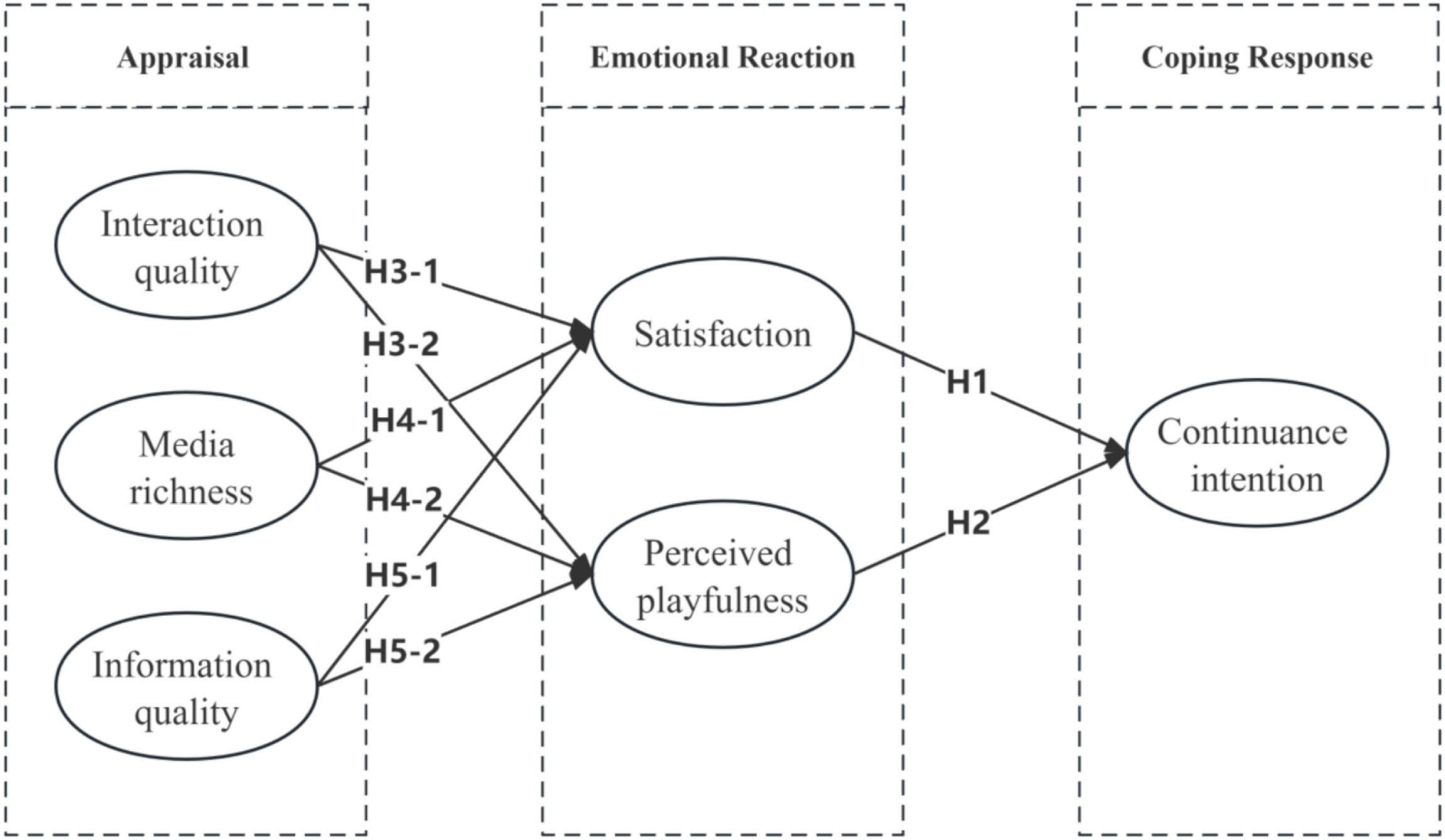
Theoretical model.

The theoretical model was shown in Figure 2.

## 4 Research methodology

### 4.1 Data collection

The main survey was executed as an anonymous cross-sectional study in China, utilizing Questionnaire Star, a professional research questionnaire software developed by Changsha Survey-Star Information Technology Co., Ltd. The questionnaire was presented in Chinese to ensure respondents’ complete understanding of each question. Questionnaire Star is a platform known for its capability to yield representative samples and is widely used in research. The survey was disseminated through various WeChat groups focused on culture and tourism, with participants informed about the possibility of receiving a random digital gift upon completion.

The survey was conducted between August 28 and September 28, 2023. Out of the 577 responses gathered, 408 were deemed valid after excluding surveys with incorrect answers to screening questions or unusually short response times. This resulted in a conversion rate of 70.70%. The demographic and other relevant information of the respondents is presented in Table 1.

**Table 1:** Respondent demographics.

Items	Category	Frequency (*N* = 408)	Percentage (%)
Gender	Male	195	47.80%
	Female	213	52.20%
Age (years)	<20	148	36.27%
	21–30	126	30.88%
	31–40	58	14.22%
	41–50	57	13.97%
	>50	19	4.66%
Education	High school or below	79	19.36%
	Junior college or Bachelor	253	62.01%
	postgraduate or above	76	18.63%
Income	<3,000	246	60.29%
	3,000–5,000	67	16.42%
	5,000–8,000	48	11.76%
	8,000–10,000	30	7.35%
	>10,000	17	4.17%

Table 1 indicates a balanced gender distribution among respondents, with 47.80% male and 52.20% female. A total of 148 (36.27%) respondents were under 20 years of age, 184 (45.10%) were 20–40 years old, and 76 (18.63%) were 41 years old or older. In terms of their education, 79 (19.36%) had a high school education or lower, 253 (62.01%) had graduated from a university or college, and 76 (18.63%) had studied in graduate school. The monthly income distribution of the respondents was as follows: 246 respondents (60.29%) reported earning less than 3,000 yuan, 67 respondents (16.42%) earned between 3,000–4,999 yuan, 48 respondents (11.76%) earned between 5,000–7,999 yuan, 30 respondents (7.35%) earned between 8,000–9,999 yuan, and 17 respondents (4.17%) earned over 10,000 yuan.

### 4.2 Survey design and measurement items

The objective of this research is to explore the determinants affecting users’ continuance intention to engage with digital museums. To examine the interplay among these factors, Partial Least Squares (PLS) methodology was employed for data analysis. The questionnaire for this study was structured into three distinct parts.

In the first part, we selected Digital DunHuang (DDH) - Cave 285, a digital museum that can be accessed online for free, for the simulation experiment. DDH is a virtual project for the preservation of Dunhuang culture, realistically reproducing over 180 historical aspects of the Mogao Caves through digital means such as VR, AR, and interactive reality. It fulfills the desire of individuals to visit and study the Mogao Caves anytime and anywhere. The choice of DDH for the simulation experiment is based on two reasons. First, since its launch in 2011, it is one of the largest digital museums in China with a mature information system. Second, by using VR and AR to create an immersive and multi-sensory setting, combined with effective communication skills, advanced technology, and innovative storytelling, it offers an engaging and unique touring experience to users. Notably, participants in the survey questionnaire are required to first access DDH - Cave 285 (see the virtual exhibition hall of Digital DunHuang: https://285.e-dunhuang.com/#/) for the simulation experience before moving on to the second part.

The second part of the questionnaire queried users on 22 items, aiming to evaluate seven potential variables: interaction quality, media richness, information quality, satisfaction, perceived playfulness, and continuance intention, as detailed in Table 2. To verify the authenticity of the responses, an additional item was included in this section, requiring participants to indicate whether they ‘support’ or ‘do not support’ a specific statement.

**Table 2:** Measurement scale.

Construct	Indicator	Description	References
Interaction quality (InQ)	InQ1	The DDH provided me with a high standard of interaction.	Pandey and Sahu (60)
	InQ2	The DDH responded quickly to my actions.	
	InQ3	The DDH responded quickly to my needs.	
	InQ4	Generally, the quality of interaction provided by DDH is very good.	
Media richness (MR)	MR1	The DDH can deliver information in a number of ways.	Otondo et al. (61), Oh et al. (62)
	MR2	The DDH allowed me to understand the symbolic meaning of the exhibits in addition to displaying them.	
	MR3	The DDH can provide me instant feedback upon my requests.	
	MR4	Overall, the DDH provide me with a wealth of information about exhibits.	
Information quality (IQ)	IQ1	The information and content provided by the DDH is easy to understand.	Chavez et al. (63), Wu et al. (14)
	IQ2	The DDH provide clear information and content.	
	IQ3	The DDH present information in the form of an appropriate interface.	
	IQ4	The information provided by the DDH is very good.	
Perceived playfulness (PP)	PP1	When interacting with the DDH, I do not realize the time elapsed.	Mathwick et al. (64), Hsu and Chiu (65)
	PP2	When interacting with the DDH, I am not aware of any noise.	
	PP3	The DDH tour is very interesting to me.	
Satisfaction (SAT)	SAT1	I am satisfied with the performance of the DDH.	Moon and Kim (34), Hsu and Chiu (65)
	SAT2	I am pleased with the experience of using the DDH.	
	SAT3	My decision to use the DDH was a wise one.	
Continuance intention (CI)	CI1	I intend to continue using the DDH rather than discontinue its use.	Bhattacherjee (16), Roca et al. (66)
	CI2	My intentions are to continue using the DDH rather than any alternative means.	
	CI3	I will frequently use the DDH to acquire knowledge in the future.	
	CI4	I highly recommend that others use the DDH.	

The final part of the questionnaire focused on gathering the respondents’ professional and demographic details, such as their education level, monthly income, age, and gender.

To guarantee the usefulness of the questionnaire, we generated all of the items based on pertinent expert feedback and previous studies. To evaluate the items, we used a five-point Likert scale (1 = “strongly disagree,” and 5 = “strongly agree”). We adopted four items to measure the interaction quality (60), and four items relating to media richness from Otondo et al. (61) and Oh et al. (62), as well as four items relating to the information quality from Chavez et al. (63) and Wu et al. (14). Also, we selected the items related to gameplay and satisfaction from Mathwick et al. (64), Hsu and Chiu (65), and Moon and Kim (34), respectively. Lastly, we measured continuance intention using four items from Bhattacherjee (16) and Roca et al. (66). Adopting scale items from previous research further enhanced this study’s reliability. In addition, item expression was refined to indicate the scope of the study. Table 2 provides the measurement items for all constructs.

Prior to the official distribution of the questionnaire, a pre-test was carried out with 30 users to validate the survey’s accuracy. These pre-test participants affirmed their comprehension of the survey items and its purpose. However, feedback from 4 users indicated a lack of clarity in the items related to interaction quality. As a result, we expanded the section on interaction quality with explanations and examples.

## 5 Result

### 5.1 Measurement model

This paper employed PLS as the chosen method for structural equation modeling, with Smart PLS v4.0.9 serving as the designated software tool to scrutinize the hypotheses formulated in the study. The PLS-SEM methodology functions by estimating relationships between latent variables through a series of regression equations. It identifes key factors that elucidate the most variation in the data and subsequently determines the relationships between these factors and the latent variables. Additionally, the technique calculates the indirect efects of these latent variables on the observed variables, providing a comprehensive understanding of the interconnections within the model (67). Although this method has been widely applied across various social science disciplines, including marketing, international management, it also has certain limitations, e.g., the theoretical foundation of measurement models, the complexity of dealing with nonlinear effects and endogeneity, and the potential for bias caused by unobserved heterogeneity, etc. These limitations require researchers to integrate theory and empirical methods when using PLS-SEM and to interpret the results with caution (68).

To ensure the reliability and validity of the measurement model, the study followed the guidelines proposed by Fornell and Larcker (69). Initially, Cronbach’s alpha was utilized to evaluate the internal consistency reliability of each construct. Table 3 indicates that, with the exception of one construct, the Cronbach’s alpha values for the constructs surpassed the recognized critical value of 0.8. Moreover, an assessment of the measurement model’s convergent validity was conducted. Table 4 shows that all but one of the loadings exceeded the benchmark of 0.7, with media richness being a close exception. The Average Variance Extracted (AVE) values ranged from 0.583 to 0.635, all above the minimum requirement of 0.5, and the composite reliability values were consistently above the recommended threshold of 0.6 (Table 3). Discriminate validity was also tested. Table 5 reveals that the highest correlation between any two constructs is 0.665, with the smallest square root AVE being 0.763. This indicates that the square root of the AVE for every construct surpassed the inter-construct correlations. In summary, the model demonstrated sufficient reliability, convergent validity, and discriminant validity.

**Table 3:** Descriptive statistics, α, composite reliability, and AVE.

Construct	Mean	SD	A	CR	AVE
Interaction quality (InQ)	3.588	0.926	0.861	0.874	0.635
Media richness (MR)	4.638	0.587	0.861	0.862	0.611
Information quality (IQ)	4.329	0.732	0.864	0.848	0.583
Perceived playfulness (PP)	4.351	0.729	0.871	0.838	0.632
Satisfaction (SAT)	4.073	0.927	0.858	0.839	0.634
Continuance intention (CI)	3.970	0.926	0.877	0.867	0.620

**Table 4:** Cross-loadings.

Construct		Factor1	Factor2	Factor3	Factor4	Factor5	Factor6
Interaction quality (InQ)	InQ4	0.840					
	InQ2	0.824					
	InQ3	0.767					
	InQ1	0.753					
Media richness (MR)	MR3		0.862				
	MR2		0.821				
	MR4		0.737				
	MR1		0.694				
Information quality (IQ)	IQ1			0.820			
	IQ4			0.766			
	IQ2			0.733			
	IQ3			0.731			
Perceived playfulness (PP)	PP2				0.813		
	PP1				0.790		
	PP3				0.782		
Satisfaction (SAT)	SAT1					0.847	
	SAT2					0.774	
	SAT3					0.766	
Continuance intention (CI)	CI1						0.813
	CI2						0.799
	CI3						0.797
	CI4						0.738

**Table 5:** Correlations and square root values of AVEs.

Construct	1	2	3	4	5	6
1. Interaction quality (InQ)	0.797					
2. Media richness (MR)	0.322	0.781				
3. Information quality (IQ)	0.262	0.533	0.763			
4. Perceived playfulness (PP)	0.235	0.452	0.665	0.795		
5. Satisfaction (SAT)	0.419	0.386	0.364	0.289	0.797	
6. Continuance intention (CI)	0.513	0.350	0.322	0.288	0.564	0.787

Off-diagonal elements are the correlation between two distinct constructs. Diagonal elements are the square root of the AVE for each construct.

### 5.2 Structural model

**Figure 3:**
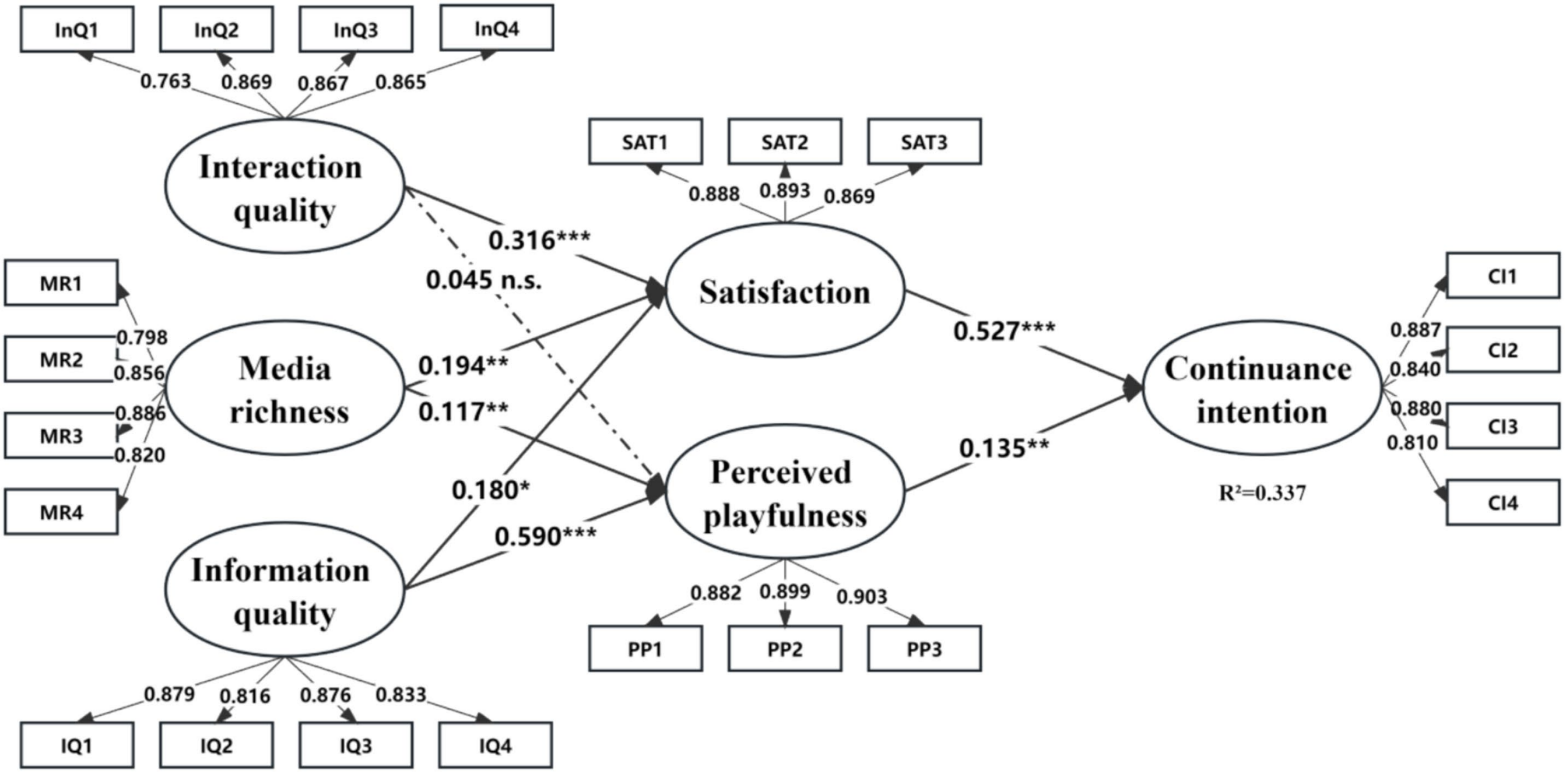
PLS results (***p < 0.001, **p < 0.01, *p < 0.05).

The results of the structural model analysis are presented in Figure 3 and Table 6. Initially, the findings indicate that both satisfaction (*β* = 0.527, *p* < 0.001) and perceived playfulness (*β* = 0.135, *p* < 0.01) have notable positive effects on users’ continuance intention, corroborating hypotheses 1 and 2. Secondly, media richness is observed to have a notable positive impact on satisfaction (*β* = 0.194, *p* < 0.01) and perceived playfulness (*β* = 0.117, *p* < 0.01), thus affirming hypotheses 4–1 and 4–2. Thirdly, information quality demonstrates a significant positive effect on both satisfaction (*β* = 0.180, *p* < 0.05) and perceived playfulness (*β* = 0.590, *p* < 0.001), supporting hypotheses 5–1 and 5–2. Lastly, interaction quality emerges as a critical determinant of satisfaction (*β* = 0.316, *p* < 0.001); however, its influence on perceived playfulness does not reach statistical significance (*β* = 0.045, *p* > 0.05), resulting in the acceptance of hypothesis 3–1 and the rejection of hypothesis 3–2.

**Table 6:** PLS results.

Hypotheses	Relationships	B	Std. Error	Result
H1	SAT - > CI	0.527***	0.046	Supported
H2	PP - > CI	0.135**	0.045	Supported
H3-1	InQ - > SAT	0.316***	0.051	Supported
H3-2	InQ - > PP	0.045 n.s.	0.037	Not supported
H4-1	MR - > SAT	0.194**	0.067	Supported
H4-2	MR - > PP	0.117**	0.044	Supported
H5-1	IQ - > SAT	0.180*	0.076	Supported
H5-2	IQ - > PP	0.590***	0.066	Supported

InQ, Interaction quality; MR, Media richness; IQ, Information quality; PP, Perceived playfulness; SAT, Satisfaction; CI, Continuance intention. ***p < 0.001, **p < 0.01, *p < 0.05; n.s. denotes not supported.

Furthermore, the study also explored the total and mediating effects of satisfaction and perceived playfulness on other variables within the research model. As shown in Table 7, user satisfaction emerges as the most significant factor on continuance intention, followed by information quality, interaction quality, perceived playfulness, and media richness.

**Table 7:** Total effects table.

	Perceived playfulness (PP)	Satisfaction (SAT)	Continuance intention (CI)
Interaction quality (InQ)	0.045 n.s.	0.316***	0.173***
Media richness (MR)	0.117**	0.194**	0.118**
Information quality (IQ)	0.590***	0.180*	0.174***
Perceived playfulness (PP)			0.135**
Satisfaction (SAT)			0.527***

***p < 0.001, **p < 0.01, *p < 0.05; n.s. denotes not support.

## 6 Discussion

This study investigates the factors that influence users’ continuance intention to use digital museums by utilizing Bagozzi’s self-regulation framework.

Firstly, consistent with previous findings (19, 22, 70), this study indicates that consumers’ continuance intention is strongly predicted by their level of satisfaction and perceived playfulness. Users with greater satisfaction or higher perceived amusement are more likely to exhibit increased rates of subsequent usage. And again in line with other research (11, 15, 21), this study showed the beneficial effects of interaction quality, media richness, and information quality on satisfaction. Users will be more happy with the system if digital museums can ensure interaction quality, information quality, and media richness.

Secondly, the study indicates a positive influence of media richness and information quality on perceived playfulness. However, the impact of interaction quality on perceived playfulness is not significant, which differs from previous studies. A possible reason is that presenting cultural relics through various channels of digital interaction technologies may lead to misunderstandings, it makes some users feel a sense of anxiety and loss of control when using the technology, which in turn affects the perceived playfulness (11). Additionally, the interaction quality perceived by users may exist only in user-system and user-content interactions, lacking user-user interactions. With the impact of social media, the diverse social needs of younger users are becoming more pronounced. Besides convenient digital systems and abundant cultural content, they are more inclined to engage in real-time communication with other users and share emotional value, a capability that is still not fully realized in digital museum experiences (71).

Moreover, the research confirms that users evaluate the interaction quality, media richness, and information quality provided by digital museums, and these evaluative factors contribute to the formation of perceived playfulness and satisfaction. Finally, perceived playfulness and satisfaction positively influence users’ continuance intention to use digital museums.

### 6.1 Theoretical implications

This work adds some noteworthy theoretical insights to the body of literature. First of all, this study advances the field of study on digital museums by offering a theoretical framework for comprehending how user satisfaction and perceived playfulness are influenced by interaction quality, media richness, and information quality, and how these factors ultimately increase users’ intention to use digital museums going forward. The findings have expanded our understanding of digital museums and identified a number of factors that impact consumers’ intention to continue using them. Meanwhile, it examines users’ continuous intentions in digital museums using Bagozzi’s self-regulation paradigm, which is a novel study to date and gives new perspectives for future research.

Secondly, the study reveals that the evaluative factors (interaction quality, media richness, and information quality) offer considerable insights into the benefits users perceive from digital museums. It establishes that elevated levels of media richness and information quality positively influence both user satisfaction and perceived playfulness, consistent with previous research. However, it is noteworthy that interaction quality exclusively affects user satisfaction.

Lastly, the study identifies user satisfaction and perceived playfulness as critical emotional reaction predictors for users’ continuance intention. The findings also demonstrate that satisfaction alone does not completely explain users’ emotional reactions. Significantly, perceived playfulness plays a critical role in adopting digital museums. Since digital museums encourage the fusion of education and entertainment, known as “edutainment” (72). Therefore, the importance of users’ perceived playfulness in the context of digital museums warrants greater acknowledgment.

### 6.2 Practical implications

Based on the above discussion, our findings provide some managerial implications for system construction of digital museums. First, in the multifaceted internet environment, digital museums should strategically utilize advanced technologies like VR, AR, and MR, rather than indiscriminately adopting the latest digital advancements. Haphazard technology stacking not only fails to increase user satisfaction but also can elevate users’ technological anxiety (73), impacting their continuance intention. Additionally, digital museums should aim for intuitive and user-friendly interactive designs, blending static visualization and dynamic interaction to cater to diverse age groups, thereby personalizing and enhancing the user experience.

Second, digital museum administrators can consider incorporating social media elements into current platforms, such as establishing online dialog systems, content-sharing platforms, and user communities, to maximize emotional engagement and foster a sense of cultural belonging to the digital museum among users (74). And, museums could explore AI projects like Meta Humans to facilitate real-time interaction with users, enriching the user experience with more engaging and enjoyable elements.

Third, the main objective of digital museums as information-dissemination systems is to maximize the information that is made available to the public. Hence, digital museums can adopt rich and engaging media forms such as “text + audio + images +3D + videos + VR interaction” to promote the integration of knowledge, science, and art. This can greatly reduce user fatigue, capture attention, elevate perceived value, and facilitate the spread of cultural information more effectively.

Fourth, information quality is pivotal in the interaction between digital museums and users. Digital museums must focus on curating high-quality cultural content to attract audiences and gain social recognition. Given that users often have specific information needs when accessing digital museums (75), providing accurate, comprehensive, and reliable information, along with tailored exhibition services based on users’ information preferences and esthetic inclinations, is essential.

At last, satisfaction is a critical determinant of users’ continuance intention. Administrators of digital museums should prioritize user satisfaction, as its development is crucial for fostering users’ continuance intention to use their services. Furthermore, the enhancement of perceived playfulness in digital museums is an integral factor in retaining and attracting a younger user base, necessitating the judicious application of technology and innovative service offerings in their operational strategy.

### 6.3 Limitations and recommendations

There are several limitations that provide direction for future research. Firstly, it employs Bagozzi’s self-regulation framework to explore factors influencing users’ continuance intention in the context of digital museums. Future research could benefit from applying different theoretical models, such as Expectation Confirmation Theory or the Technology Acceptance Model, to provide a more comprehensive understanding of users’ continuance intentions. Secondly, the empirical study’s sample predominantly consists of respondents under 30 years of age, which may limit the generalizability of the findings. Future studies should consider a more diverse age range to enhance the applicability of the model across a broader demographic spectrum. Thirdly, the current study focuses on the continuance intention to use digital museums without examining actual user behavior. Future research could explore this aspect through longitudinal studies, potentially offering richer insights into how intentions translate into actual user behavior within the digital museum context.

## Data Availability

The raw data supporting the conclusions of this article will be made available by the authors, without undue reservation.
